# Prevalence of iron deficiency anemia and beta thalassemia carriers among relatives of beta thalassemia patients in Nile Delta region, Egypt: a multicenter study

**DOI:** 10.1186/s42506-021-00088-9

**Published:** 2021-10-11

**Authors:** Mohamed R. El-Shanshory, Laila M. Sherief, Hoda M. Hassab, Seham M. Ragab, Sohier Yahia, Ahmed K. Mansour, Adel S. Ahmed, Said H. Abdou, Amal M. Helmy, Mona M. Watany, Ahmed M. Gad ALllah, Myriam A. Guindy, Zeinab I. Mourad, Mohamed A. Soliman, Reham M. El-Farahaty, Faeza El-Dahtory, Ahmad Darwish, Suzy Abd Elmabood, Ibrahim A. Kabbash, Shimaa M. Saied

**Affiliations:** 1 Pediatric Department, Faculty of Medicine, Tanta University, Tanta, Gharbia Egypt; 2 Pediatric Department, Faculty of Medicine, Zagazig University, Zagazig, Egypt; 3 Pediatric Department, Faculty of Medicine, Alexandria University, Alexandria, Egypt; 4 Pediatric Department, Faculty of Medicine, Menoufia University, Shibin el Kom, Egypt; 5 Pediatric Department, Faculty of Medicine, Mansoura University, Mansoura, Egypt; 6 Clinical Pathology Department, Faculty of Medicine, Tanta University, Tanta, Egypt; 7 Clinical Pathology Department, Faculty of Medicine, Zagazig University, Zagazig, Egypt; 8 Clinical and Chemical Pathology Department, Faculty of Medicine, Alexandria University, Alexandria, Egypt; 9 Clinical Pathology Department, Faculty of Medicine, Menoufia University, Shibin el Kom, Egypt; 10 Clinical Pathology Department, Faculty of Medicine, Mansoura University, Mansoura, Egypt; 11 Consultant of Biochemistry, Genetic Unit, Children Hospital, Mansoura University, Mansoura, Egypt; 12 Public Health and Community Medicine Department, Faculty of Medicine, Tanta University, Tanta, Egypt

**Keywords:** Screening, Thalassemia carriers, Mid Delta, Egypt

## Abstract

**Background:**

Screening of β thalassemia among close relatives is more feasible in highly prevalent countries with limited resources. The purpose of this study is to determine the prevalence of β thalassemia carriers and iron deficiency anemia among relatives of β thalassemia patients in Mid Delta, Egypt.

**Methods:**

This is a cross-sectional multi-center study conducted on 2118 relatives of patients with β thalassemia from different Egyptian governorates in the Mid Delta region. They were subjected to history taking with precise determination of geographic location, general examination, and the following investigations: complete blood counts, serum ferritin for those who showed microcytic hypochromic anemia, and high-performance liquid chromatography for those who were not diagnosed as iron deficiency anemia.

**Results:**

The total prevalence of iron deficiency anemia among close relatives of confirmed β thalassemia patients in the Nile Delta region was 17.19%. The highest prevalence of iron deficiency anemia (45.05%) was reported in Al-Gharbia Governorate, followed by Al-Menoufia Governorate (21.67%), and the lowest prevalence was that of Al-Sharkia Governorate (4.91%). The differences were highly statistically significant (*p* < 0.001). β thalassemia carrier prevalence rate in the studied relatives was 35.84%, with the highest prevalence detected in Al-Sharkia Governorate (51.32%), followed by Kafr-Alsheikh and Al-Dakahilia Governorates (41.78%, 37.13%) respectively, while Al-Menoufia Governorate had the lowest prevalence rate (25.00%). These differences were also highly statistically significant (*p* < 0.001).

**Conclusion:**

More than one-third of relatives of patients with β thalassemia are carriers of the disease, while 17.19% suffer from iron deficiency anemia. This study demonstrates the importance of tracing the high number of beta thalassemia carriers among relatives of patients with β thalassemia in Egypt.

## Introduction

Thalassemia is an autosomal recessive common genetic disorder throughout the world [1]**.** Almost 70,000 infants are born with β thalassemia worldwide each year [2]. Consanguineous marriages, a high fertility rate, a high birth rate, a low educational level, and early marriages, combined with an unawareness of the thalassemia problem, make developing countries to have a high number of transfusion-dependent thalassemia children in the world [3]. In general, patients with thalassemia major place a considerable burden on their families and health authorities [4].

Several countries have implemented national prevention programs, including public awareness and education, carrier screening and counseling, and information on the prenatal and pre-implantation diagnosis of the disease [5]. Carrier screening has had great success, leading to a decline in the birth rate of thalassemia major in some countries [5]. It has been estimated that one thousand children out of 1.5 million live births are born each year suffering from thalassemia in Egypt [6]. It is reported that the carrier rate in Egypt is between 9 to 10% of the population [7]**.**

In Egypt, despite the high prevalence of β thalassemia carriers and the growing number of patients born each year, there is no national thalassemia prevention program [8]. Few studies were performed to assess the carrier rate of β thalassemia [9, 10]. Selective screening approach within the families suffering from thalassemia is ideal and more feasible in highly prevalent regions with limited resources. The aim of our study was to determine β thalassemia carriers, in addition to iron deficiency anemia, individuals among relatives of β thalassemia patients, especially in population crowded regions, for raising the awareness of the problem among this high-risk population.

## Methods

### Study design and setting

The current study is a cross-sectional multi-center study conducted on 2118 relatives of patients with β thalassemia from different Egyptian governorates in the Mid Delta region (Al-Gharbia, Al-Dakahlia, Al-Menuofiea, Al-Sharkhia, Kafer el-Sheikh, Al-Beheira, and Alexandria).

### Participants

The study participants represented respondents who were eligible and agreed to participate in the study from the relatives (2nd, 3rd, and 4th degree) of all known β thalassemic children attending hematology/oncology clinics at Tanta, Zagazig, Mansoura, Menuofia, and Alexandria Universities’ Hospitals during the 48 months of the study (from 2016-2020). According to Egyptian law, brothers and sisters are considered second degree relatives. Grandparents, grandchildren, aunts, uncles, nieces, and nephews are considered third degree relatives, and cousins are considered fourth degree relatives. The study population included 963 male and 1155 female. The excluded relatives were those with other hemolytic anemia, relatives of known α thalassemic patients, and parents of β thalassemic patients.

### Data collection

All the individuals included in the study were subjected to full history taking, thorough clinical examination, and the following investigations: complete blood count (CBC) by automated ABX PENTRA XL80 device. The cut-off level for hemoglobin used to classify subjects into anemic and non-anemic; the hemoglobin level less than 11 g/dl in the age group between 6 and 12 years [11], and hemoglobin level < 13g/dl in male and < 12 g/dl in female in the age group more than 12 years [12]. Mean corpuscular volume (MCV) of less than 80 fl and/or mean corpuscular hemoglobin (MCH) of less than 27 pg are generally used as cut-off points for further screening with serum ferritin to exclude iron deficiency anemia. Serum ferritin less than 15 ng/ml is considered diagnostic of iron deficiency [13]. Blood samples of participants with normal serum ferritin were analyzed by high performance liquid chromatography (HPLC) for quantitative estimation of an elevated HbA2 level, using the hemoglobin analyzer ARKRAY ADAMS A1C HA-8180T (Japan) device. HbA2 < 3.5% is considered diagnostic of β thalassemia carrier state [14]**.**

#### Sample collection and storage

Five milliliters of whole blood were collected from every subject into three vacutainer tubes. The first tube containing EDTA was used as an anticoagulant for a complete blood picture (CBC) and 2 mm into a second tube for high performance liquid chromatography (HPLC). The third tube was used for serum ferritin measurement for participants with hypochromic microcytic anemia.

### Statistical analysis of data

The collected data were coded, verified for completeness, recorded in a Microsoft Excel master sheet, and then statistically analyzed utilizing the SPSS™ (Statistics Program for Social Studies) software version 25 produced by IBM, Chicago, IL, USA. Means and standard deviations were used to describe numerical values, while frequencies were used for categorical ones.

The Chi-square test was used to test the differences between categorical variables, while the Mann-Whitney *U* test was used for independent quantitative variables. *P* value was considered significant at < 0.05 and < 0.001 for highly significant results.

## Results

**Fig. 1:**
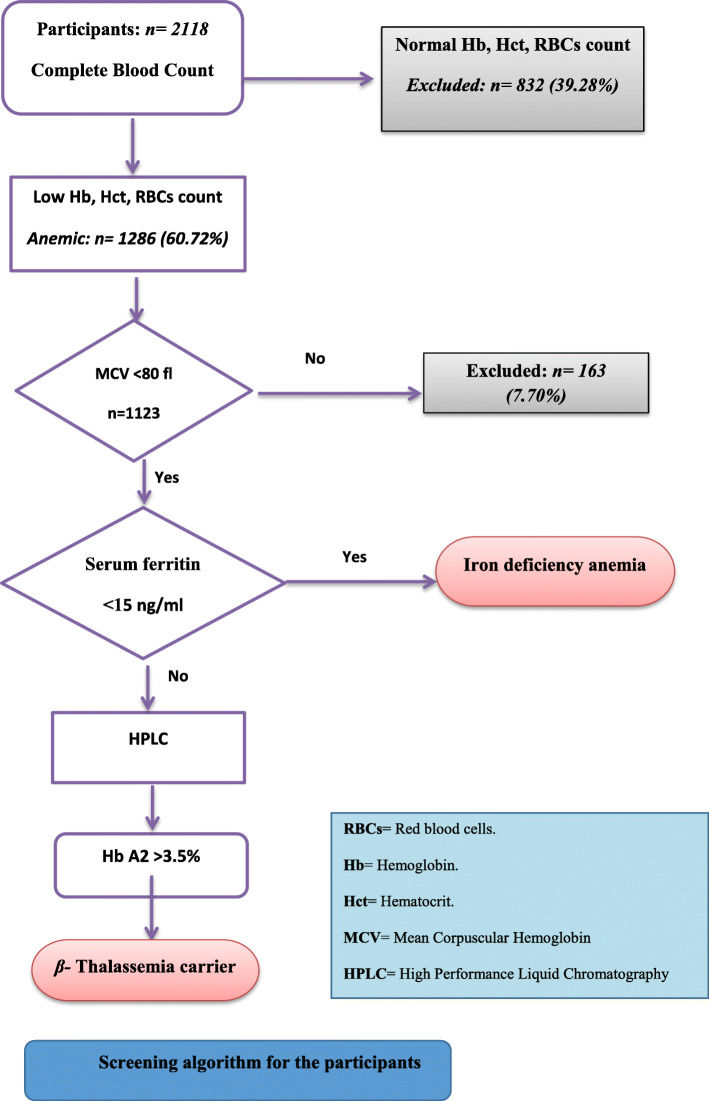
Screening algorithm for the participants

Figure 1 shows the screening algorism among the close relatives of patients with confirmed β thalassemia. The total number of participants in this study was 2118 children. Males constituted 936 participants (45.47%) while 54.53% (1155) were females. Participants with hypochromic microcytic anemia constituted 1123 (53.02%).

Table 1 illustrates the prevalence of iron deficiency anemia and β thalassemia carriers among the study participants in relation to the governorate of residence. The total prevalence of iron deficiency anemia among close relatives of confirmed β thalassemia patients in the Nile Delta region was found to be 17.19%. The highest prevalence of iron deficiency anemia (45.05%) was reported in Al-Gharbia Governorate, followed by Al-Menoufia Governorate (21.67%) and the lowest prevalence was that of Al-Sharkia Governorates (4.91%), these differences were highly statistically significant (*p* < 0.001). On the other hand, the total carrier prevalence rate in the studied relatives was 35.84%, with the highest prevalence detected in Al-Sharkia Governorates (51.32%), followed by Kafr-Alsheikh Governorate (41.78%), and Al-Dakahilia (37.13%). Al-Menoufia Governorate had the lowest prevalence rate (25.00%). These differences were also found highly statistically significant (*p* < 0.001).

**Table 1:** Prevalence of iron deficiency anemia and β thalassemia carriers among studied relatives in Mid Nile Delta Egyptian Governorates (2016-2020)

Governorate	Total examined	Iron deficiency anemia (Group 1)		β thalassemia carrier (Group 2)	
	*N*	*N*	%	*N*	%
**Al-Gharbia**	182	82	45.05	56	30.77
**Al-Menoufia**	300	65	21.67	75	25.00
**Al-Sharkia**	265	13	4.91	136	51.32
**Al-Dakahilia**	668	100	14.97	248	37.13
**Al-Beihera**	219	22	10.05	70	31.96
**Alexandria**	326	69	21.17	108	33.13
**Kafr-Elsheikh**	158	13	8.23	66	41.78
**Total**	2118	364	17.19	759	35.84
***χ***^***2***^	154.32			50.36	
***P***	< 0.001^**^			0.001**	

**Highly significant (*P* < 0.01)

Table 2 demonstrates a comparison of blood indices between β thalassemia carriers and iron deficiency anemia of the studied relatives. Only hemoglobin concentration showed no significant difference between the two groups (*p* = 0.152). The total number of red blood corpuscles among group 2 (5.28 ± 0.63) × 10^6^/mm^3^ was significantly higher than that recorded for children with the group 1 (3.74 ± 0.56) × 10^6^/mm^3^ (*p* = 0.001). The mean hematocrit percentage in group 2 (33.31 ± 4.09) % was significantly higher than that of group 1 (32.7 ± 3.71) (*p* = 0.001). Besides, mean corpuscular volume was significantly higher among group 1 (67.83 ± 7.21) fl compared to group 2 (62.42 ± 6.37) fl (*p* = 0.001). The same applies for MCH, 22.70 ± 2.81 versus 20.32 ± 2.21 pg (*p* = 0.001); MCHC, 33.94 ± 2.32 g/dl versus 32.83 ± 2.31 g/dl (*p* = 0.001).

**Table 2:** Complete blood count of β thalassemia carriers and iron deficiency anemia participants in Mid Nile Delta Egyptian Governorates (2016-2020)

Variable		Iron deficiency anemia (Group 1)	β thalassemia carrier (Group 2)	MW^#^	***P***
**Red blood corpuscles (X10**^**6**^**/mm**^**3**^**)**	*Mean ± SD*	3.74 *±* 0.56	5.28 *±* 0.63	13.45	0.001**
	*Range*	2.30-4.96	4.00-6.60		
	*Median*	3.80	5.28		
**Hemoglobin (g/dl)**	*Mean ± SD*	10.60 ± 1.21	10.95 *±* 1.20	1.43	0.152
	*Range*	6.60-12.90	9-15.40		
	*Median*	10.80	10.90		
**Hematocrit value (%)**	*Mean ± SD* *Range* *Median*	32.7 *±* 3.71 18.50-39.7 32.70	33.31 *±* 4.09 20.20-44.90 33. 10	2.34	0.019**
**Mean corpuscular volume (fl)**	*Mean ± SD*	67.83 ± 7.21	62.42 *±* 6.37	7.70	0.001**
	*Range*	45-79	45.0-79.0		
	*Median*	69.00	62.00		
**Mean corpuscular hemoglobin (pg)**	*Mean ± SD*	22.70 *±* 2.81	20.32 ± 2.21	7.20	0.001**
	*Range*	14.60-27	15.20-27.20		
	*Median*	22.75	20.10		
**Mean corpuscular hemoglobin concentration (g/dl)**	*Mean ± SD*	33.94 *±* 2.32	32.83 *±* 2.31	3.95	0.001**
	*Range*	29.50-38.20	26.80-37.80		
	*Median*	34.00	32.30		
**Red cell distribution width (%)**	*Mean ± SD*	16.89 ± 2.28	15.30 *±* 2.18	5.93	0.001**
	*Range*	11.90-21.30	11.00-18.40		
	*Median*	17.4	16.00		

*SD* standard deviation

^#^MW: *Z* value of Mann-Whitney *U* test

**Highly significant (*P* < 0.01)

Lastly, group 1 had a significantly higher mean red cell distribution width, 16.89 ± 2.28%, compared to 15.30 ± 2.18% for group 2 (*p* = 0.001).

Table 3 shows the comparison of serum ferritin and HbA2% between relatives who are β thalassemia carriers and those with iron deficiency anemia. Serum ferritin shows a significantly lower mean of 8.20 ± 3.78 ng/ml among group 1 than 68.73 ± 49.33 ng/ml among group 2 (*p* = 0.001). In comparison, the group’s 2 hemoglobin A2% was significantly higher (4.55 ± 0.5) % compared to the mean of 2.54 ± 0.49% detected among group 2 (*p* < 0.001).

**Table 3:** Serum ferritin and HbA2 of β thalassemia carriers and iron deficiency anemia participants Mid Nile Delta Egyptian Governorates (2016-2020)

Variable		Iron deficiency anemia (Group 1)	β- thalassemia carrier (Group 2)	MW^#^	*P*
**Serum ferritin (ng/ml)**	*Mean ± SD*	8.20 *±* 3.78	68.73 ± 49.33	23.35	0.001^**^
	*Range*	1.00-14.90	15.50-349		
	*Median*	6.85	55.00		
**Hemoglobin A2%**	*Mean ± SD*	2.54 ± 0.49	4.50 ± 0.59	23.36	0.001^**^
	*Range*	1.30-3.40	3.60-6.99		
	*Median*	2.60	4.500		

*SD* standard deviation

^#^MW: *Z* value of Mann-Whitney *U* test

**Highly significant (*P* < 0.01)

## Discussion

Symptomatic β thalassemia syndromes constitute a significant public health problem in Egypt; the high prevalence of beta thalassemia carriers, combined with a growing number of newly born cases, underscores the critical significance of developing a beta thalassemia prevention program in Egypt [15]. Prevention by carrier detection is needed in populations with a high incidence of the disease, such as Egypt. Improving public awareness and mandatory premarital screening for carrier detection are essential to offer prenatal diagnosis and genetic counseling for high-risk couples [15].

Various approaches of carrier screening programs were conducted in several countries. They include general population screening, high-risk group screening, antenatal screening, and cascade screening or extended family screening [16]. As an autosomal recessive disease, together with the high rate of consanguineous marriages in our country, the expected highest prevalence of β thalassemia carriers will be among the patient’s relatives who are a good target for screening. This type of screening may offer an alternative to population screening for identifying present and future couples at risk for producing affected children [17].

The current study involved 2118 relatives of patients with β thalassemia from different Egyptian governorates in the Mid Delta region. Microcytic hypochromic anemia was diagnosed in 53.02% of the studied groups. Carrier detection in this study was based upon the presence of microcytic hypochromic anemia, normal serum ferritin level together with HbA2 level of < 3.5% [14].

β thalassemia carrier state constituted the majority among children with microcytic hypochromic anemia (67.59%), with a prevalence rate of 35.84% among the studied relatives of the patients. This is about 3-4 times higher than the estimated carrier rate of 9-10% in the general population [7]. This result supports what was previously reported that β thalassemia carriers are more prevalent in siblings of thalassemia major than the normal population [18]. Most of the previous studies included general population and pregnant women screening, with few that included relatives of thalassemia patients. To our knowledge, there is no published data about similar studies in Egypt.

Our estimated prevalence of β thalassemia trait (βTT) in relatives of patients is nearly similar to what was reported in Rawalpind, Pakistan (31%) by Ahmed et al. [19] while it is higher than the prevalence reported by Gorakshakar and Colah (21.9%) in extended family screening in India [17]. On the other hand, it is lower than what was found in other studies done in Faisalabad (44.4%) [20], Kota (48.76%) [18], Bandung (59.6%) [21], Karachi (62.2%) [22], Bhopal (76%) [23], and North India (76.92%) [24]. This discrepancy could be explained by the differences in general prevalence rate in the geographic areas involved, selection criteria in each study (siblings only or extended family, the number of included individuals), and genetic heterogeneity of thalassemia gene.

In this study, the highest carrier prevalence rate was detected in Al-Sharkia Governorates and followed by Kafr-Alsheikh Governorate, Al-Dakahilia, while Al-Menoufia Governorate had the lowest prevalence rate. This variation between localities might be related to the different rate of consanguineous marriage in different Egyptian communities. In societies where the majority of couples are unrelated, genes for recessive disorders usually run in families for many generations without manifesting through the birth of an affected child. By contrast, gene variants are trapped within extended family members [19]. Thus, in communities where consanguinity is evident, one can identify even more carriers [16]. It was reported that the cascade screening result was more impressive in a small location.

For example, in Sardinia, by analyzing 11% of the population, more than 90% of the “at risk” couples were detected [25]. An affected child is a predictor of high genetic risk, and an extended family study may discover several carriers and couples at risk before marriage or reproduction [17]. Family studies provide a highly effective risk detection approach. Population screening is less effective, but carrier follow-up will detect elevated risk prior to any affected child’s birth. Moreover, the perfect approach is to provide both family studies and premarital or antenatal screening for the relatives of affected children [19]**.**

Worldwide, beta thalassemia trait (βTT) and iron deficiency anemia (IDA) are the two most frequent causes of microcytic anemia [26]*.* The differentiation between IDA and βTT is of prime importance, especially in these high-risk groups for being βT carriers [27]. Iron deficiency anemia is of high prevalence among Egyptian children. In the study performed by El-Beshlawy et al. [9], about one-third of the studied population in upper & lower Egypt had IDA based on the presence of microcytic hypochromic anemia with serum ferritin level below 15 ng/ml. In the present study, IDA was diagnosed in 17.19% of the participants. Though lower than what was estimated in the general population, IDA is prevalent among relatives of thalassemia patients and should be searched for and adequately treated. The highest prevalence of IDA (45.05%) was reported in Al-Gharbia Governorate, followed by Al-Menoufia Governorate (21.67%), and the lowest prevalence was that of Al-Sharkia Governorates (4.91%). The significant difference between different governorates could be related to the involvement of rural areas in governorates with a high prevalence of IDA, lower-income families with limited access to iron-rich foods, and inefficient utilization of available micronutrients as a result of infectious diseases, particularly helminthic infections [28]**.**

Comparing the hematological parameters of relatives with βTT and those with IDA, no significant difference was found regarding Hb level. In contrast, βTT relatives had a significantly higher RBCs count, with significant lower MCV, MCH, MCHC, and RDW than IDA. Red blood cell (RBC) count is known to be increased in both thalassemia patients and carriers. It is considered a useful diagnostic adjunct because thalassemia has microcytic anemia, increasing the RBCs number. In contrast, other causes of microcytic anemias, including iron deficiency anemia and anemia of chronic disease, are typically associated with a proportional decrease in the RBC number [29]**.**

In accordance with our results, a high RBC count among BTT was reported by Demir et al. [30]**,** Vehapoglu et al. [27], and Jameel et al. [31]. Demir et al. [30] reported that RBC count is one of the most reliable discrimination indices in differentiation between βTT and IDA, with 90% of the patients were correctly identified with RBC count. The majority of β thalassaemia carriers have reduced MCV and MCH levels in the standard complete blood examination (FBE) [31]. Low MCV is the key indicator for diagnosis and screening for thalassemia. It was suggested that an MCV of < 72 is maximally sensitive and specific for the presumptive diagnosis of thalassemia [32].

The red cell distribution width (RDW) index reflects the heterogeneity in the size distribution of erythrocytes, measuring the coefficient of variation around MCV [33]. It was found to be the most reliable index evaluated for discrimination between βTT and IDA, with 100% sensitivity and 92.6% specificity. This index can be used to efficiently screen patients with microcytosis for further hematologic studies to confirm β thalassemia [34].

Normal RDW among βTT was also reported by other studies [18, 35, 36]. So, microcytosis accompanied by a high RBC count and normal RDW is highly suggestive of BTT [36]. A definitive differential diagnosis between βTT and IDA is based on HbA2 electrophoresis, serum iron, and ferritin levels [37]. In this regard, relatives of thalassemia patients with βTT have a significantly higher level of HbA2 with significantly higher serum ferritin levels than those diagnosed as IDA. HbA2 levels (> 3.5%) are the most significant parameter for identifying beta thalassemia carriers [28]. In this study, those with βTT have HbA2 ranged between 3.60-6.99% with a mean value of 4.55 ± 0.59%. In similar studies, the mean HbA2% levels were 11.93 ± 0.5% [21], 4.99 ± 0.64% [16], and 5.24 ± 1.14% [18].

### Limitations of the study

An important limitation is the unavailability of molecular diagnosis of thalassemia and genetic screening to determine the genetic profile for individuals to identify carriers. Besides, carrier identification by premarital and/or early antenatal thalassemia screening is not mandatory and is not commonly used in Egypt. As the current state of thalassemia reflects the increasing need for national preventive program to reduce morbidity and mortality associated with this disease, particularly given the country’s limited resources and that thalassemia prevention is cost-effective. We recommend initiating a national program for extended family screening of relatives with beta thalassemia patients as a preparatory and promising step to establish a national prevention program utilizing the data collected from the screening.

## Conclusion

Thalassemia carriers have characteristic hematological parameters with mild anemia with microcytic hypochromic RBCs, increased red cell counts and normal RDW that could help screening and together with HbA2 quantification by Hb electrophoresis or HPLC, βTT can be easily diagnosed and screened for in high-risk groups like family members and relatives of thalassemia patients. Family screening program by screening for microcytic hypochromic anemia with increased RBCs reduced MCV; MCH and normal RDW is a valuable and feasible alternative to the population screening for detecting family members at risk of carrier state of βTT. Those can be further identified by measuring HbA2.

## Data Availability

The research data is available upon a reasonable request to the corresponding author.

## Abbreviations

BTT: β thalassemia trait
CBC: Complete blood count
EDTA: Ethylenediaminetetraacetic acid
FBE: Full blood examination
Hb: Hemoglobin
HPLC: High performance liquid chromatography
IDA: Iron deficiency anemia
MCH: Mean corpuscular hemoglobin
MCHC: Mean corpuscular hemoglobin concentration
MCV: Mean corpuscular volume
NTDT: Non-transfusion-dependent β thalassemia
RBCs: Red blood cells
RDW: Red cell distribution width
SPSS: Statistics Program for Social Studies
TDT: Transfusion-dependent β thalassemia
